# Disparities in Potentially Preventable Emergency Department Visits for Children with Asthma among Asian Americans, Pacific Islanders, and Whites in Hawai‘i

**DOI:** 10.3390/ijerph18137096

**Published:** 2021-07-02

**Authors:** Olivia Uchima, Deborah A. Taira, Hyeong Jun Ahn, So Yung Choi, May Okihiro, Tetine Sentell

**Affiliations:** 1 Office of Public Health Studies, University of Hawai‘i at Manoa, 1960 East-West Road, Honolulu, HI 96822, USA; tsentell@hawaii.edu; 2 Daniel K. Inouye College of Pharmacy, University of Hawai‘i at Hilo, 677 Ala Moana Boulevard, Honolulu, HI 96813, USA; dtjuarez@hawaii.edu; 3 Department of Quantitative Health Sciences, University of Hawai‘i at Manoa, 651 Ilalo Street, Medical Education Building, Honolulu, HI 96813, USA; hjahn@hawaii.edu (H.J.A.); soyung@hawaii.edu (S.Y.C.); 4 Department of Pediatrics, John A. Burns School of Medicine, University of Hawai‘i at Manoa, Honolulu, HI 96813, USA; okihirom@hawaii.edu

**Keywords:** hospitalization, costs, asthma, child, adolescent, Asians, Pacific Islanders, Native Hawaiians

## Abstract

The cost burdens of potentially preventable emergency department visits for pediatric asthma were estimated for Asian Americans, Pacific Islanders, and Whites using Hawai‘i statewide 2015–2016 data. The cost burden of the 3234 preventable emergency department visits over the study period was over $1.9 million. Native Hawaiians had the largest proportion (36.5%) of all preventable emergency department visits and accounted for the highest costs for the two years at $709,698. After adjusting for other factors, costs for preventable pediatric-asthma-related emergency department visits differed significantly by age, insurance provider, and island of residency. Reducing potentially preventable emergency department visits would not only improve health disparities among Native Hawaiians compared to other racial or ethnic populations in Hawai‘i, but could also generate cost savings for public and private insurance payers.

## 1. Introduction

Hawai‘i is a multicultural state with two-thirds (66%) of the population being Asian American or Pacific Islander [1]. About 34% of Hawai‘i’s children are Native Hawaiian, which is the largest racial or ethnic population as compared to the other major races or ethnicities [2]. Native Hawaiians are not only Hawai‘i’s indigenous people, they are one of the fastest growing racial or ethnic groups in the United States (US) [3]. What is concerning is that Native Hawaiians experience higher rates of chronic health disparities, including asthma, stroke, hypertension, diabetes, and heart disease, which may occur during childhood [3,4]. Native Hawaiian children also have a higher asthma prevalence than any other racial or ethnic group in Hawai‘i [5]. In addition, Native Hawaiian children may face sociocultural challenges, other health-related comorbidities, and decreased access to high quality health care [6]. These challenges affect asthma management, which can result in increased asthma severity, outcomes, and utilization of acute health care services.

The Agency for Health Care Research and Quality (AHRQ) reported childhood asthma as an indicator of potentially preventable pediatric emergency department (PPPED) visits [7]. The AHRQ Prevention Quality Indicators defines PPPED visits as those that could most likely be prevented through effective chronic care management, timely access to high quality, primary care services, or improvements to social determinants of health [7,8]; however, not every pediatric asthma ED visit is considered potentially preventable [8,9]. Specific age and comorbidities, such as allergic rhinitis among children aged 2–17 years, also help to define PPPED visits for asthma [10]. Factors associated with PPPED visits have been less studied, but research has shown minority race or ethnicity, Medicaid insurance, distance to health care professionals or facilities (i.e., rural residence), and comorbidities are typically correlated with potentially preventable pediatric hospitalizations [11,12].

Reducing the frequency of PPPED visits, such as asthma exacerbations, has the potential to save lives, as well as money [13]. Medicaid is moving from a volume-based system towards a value-based payment system. Traditionally, medical providers were incentivized based on the quantity of services provided (i.e., fee-for-service); however, there has been a paradigm shift to value-based services that focuses on the quality of the health care provided [13]. As such, this is a topic of significant interest for policy makers and decision makers in health care administration to create “targeted solutions to improve the value and quality of care delivered” for children with asthma and to reduce poor quality of care [14].

Asthma education programs are critical to not only improve asthma morbidity among children but also to reduce asthma-related health outcomes. These programs highlight the importance of avoiding environmental and dietary triggers as well as adherence to prescribed medication [14]. While to our knowledge there are no studies that have specifically examined the effectiveness of these interventions for reducing PPED visits, asthma education programs have demonstrated improvements in self-management, asthma knowledge, asthma symptoms, and self-efficiency; increased compliance with medication and quality of life; and reductions in ED visits and hospitalizations [15,16,17,18,19,20]. As such, data are needed to understand the scope of the issue in order to prioritize funding efforts that focus on the development of asthma education programs to reduce PPPED visits.

### 1.1. National Data

Every year in the US, approximately 4 million children have an asthma exacerbation event, defined as breathlessness, coughing, wheezing, and chest tightness. These events often require immediate medical care and account for more than 1.8 million ED visits annually [21,22]. In 2010, an evaluation of state Medicaid programs estimated asthma-related ED visit costs at $272 million [21]. From 2001 to 2010, the annual rate of PPPED visits among children with asthma rose by 16% from 531,374 visits in 2001 to 615,958 in 2010 [23].

Racial or ethnic disparities exist in PPPED visits, with a disproportionate percentage of Black or Hispanic children having more frequent ED visits compared to Whites [14]. However, these estimates have not disaggregated Asian and Native Hawaiian populations, which make it challenging to fully recognize the cost and health burdens on these vulnerable populations [14]. The disaggregation of Asian and Native Hawaiian populations into subgroups are important because racial or ethnic disparities may exist and be overlooked. For example, Native Hawaiians, Filipinos, and Japanese adults have higher potentially preventable hospitalization rates for diabetes and cardiovascular disease than other Asian subgroups (e.g., Chinese) and compared to Whites [24]. Additionally, these racial or ethnic groups have different demographic characteristics, such as household size and household income, which are potential risk factors for asthma.

Previous studies have estimated the economic burden of asthma for potentially preventable hospitalizations at the national and state levels; however, none are ED-specific [14,25,26,27,28,29]. This is imperative, as the ED may be the only place of care for children with asthma who have limited access to medical providers and for parents or caregivers to be educated in asthma management [21]. Additionally, none of this research has been performed in Hawai‘i. These issues are of particular importance because Native Hawaiians who reside in Hawai‘i experience disparities in potentially preventable hospitalizations for other chronic diseases, as well as disparities in socioeconomic status and other social determinants of health [24,30]. They also have disproportionately higher costs for preventable hospitalizations for cardiovascular disease and diabetes [24].

### 1.2. Study Objective

Knowing that asthma-related ED visits among children can be prevented, the focus of this study was two-fold. The study aimed to: (1) estimate whether the total direct costs for asthma-related services among children with PPPED visits differ across each major race or ethnicity in Hawai‘i after the adjustment for other factors; (2) examine whether the average numbers of visits for children with at least one PPPED visit differ among each major race or ethnicity in Hawai‘i. Knowledge of the scale and economic burden among vulnerable populations for costly health system outcomes of childhood asthma provides policy makers and decision makers in health care administration a better understanding of the scale, magnitude, and implications of the problem. It may also support the development of targeted interventions to better address asthma-related disparities in Hawai‘i and in other locations.

## 2. Methods

### 2.1. Data Source

A retrospective study was conducted using the Hawai‘i Health Information Corporation (HHIC) ED data from January 2015 through December 2016. Data were obtained from 23 private operating acute care hospitals in Hawai‘i during the study period. The HHIC database collected information from all but one of the hospital-based EDs and all of the acute care hospitals in the state [31]. The HHIC has detailed discharge data from all non-federal hospitalizations and ED visits by all payers, and includes race or ethnicity, insurance provider, sex, age, location of hospital, length of stay, geographic residence of patient, as well as International Classification of Diseases, Ninth Revision, Clinical Modification (ICD-9) and International Classification of Diseases, Tenth Revision, Clinical Modification (ICD-10) primary and secondary diagnoses and procedure codes [32]. Both ICD-9 and ICD-10 codes are included because the transition from ICD-9 to ICD-10 codes started in October 2015.

### 2.2. Study Population

**Figure 1:**
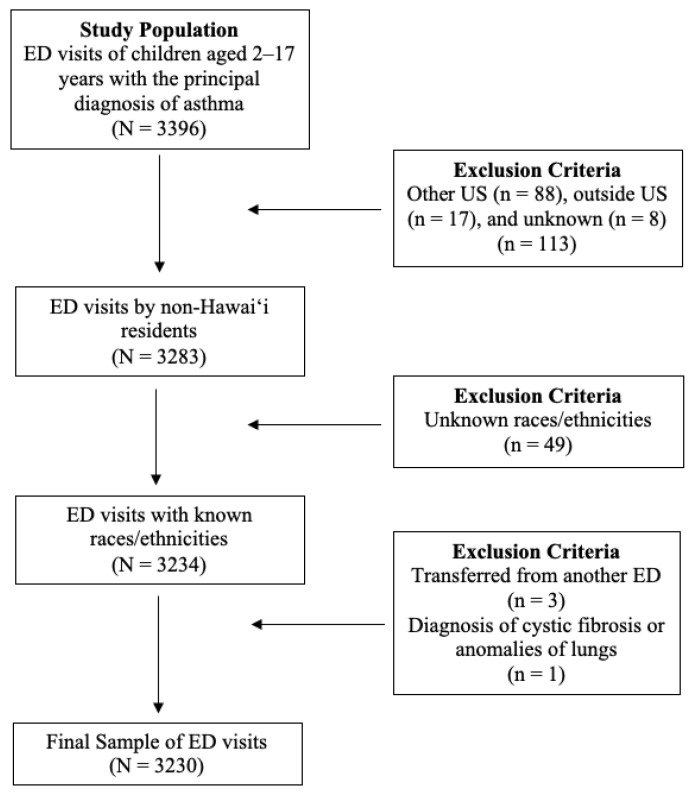
Flowchart of Exclusion Criteria.

All ED visits of children who were between the ages of 2 to 17 years old with the principal diagnosis of asthma were considered. As this study specifically focused on the racial or ethnic populations of Hawai‘i, non-Hawai‘i residents and records lacking race or ethnicity data were also excluded. Additionally, transfers from another ED facility and children with any listed ICD-9 or ICD-10 diagnosis codes for cystic fibrosis and anomalies of the lungs were excluded to meet AHRQ potentially preventable pediatric ED visits for asthma specifications [8,9]. More detail about specific ICD-9/ICD-10 diagnosis codes for cystic fibrosis and anomalies of the respiratory system are provided by AHRQ [8,9]. A total of 3230 ED visits were considered (see Figure 1).

The HHIC data included a master patient identification variable, which was used to track individuals across all hospitals in Hawai‘i. An individual’s unique identification number assigned within the database was used to identify multiple ED visits. The number of children or unique individuals was considered in order to confirm multiple ED visits among particular children from one racial or ethnic group were not driving health disparities. The analyses were conducted using the first, last, and a random ED visit for each individual. The results across each visit did not change significantly, so the first ED visit for each individual was used in the main study analyses; however, to identify the full cost burden and understand the scope of the financial impact of this problem, when estimating the cumulative cost, all ED visits by individuals were included.

### 2.3. Measures

Potentially preventable ED visits: AHRQ specifications were followed to measure PPPED visits with the principal diagnosis of asthma. PPPED visits for asthma include only children aged ≥2 years with one of the 14 principal ICD-9 or 13 ICD-10 diagnosis codes. See Table 1 for the following ICD-9 and ICD-10 codes that were included [8,9].

Costs: Costs were estimated from hospital charges using established ED cost/charge ratios (CCRs) available by the American Hospital Directory (AHD) for each hospital in this dataset [33]. The following formula was used to compute the estimated cost: (ED Charges * ED CCR) = Estimated cost. Charges of each ED visit was based on the amount that was billed by the hospital. These charges are determined based on the insurance payer, the principal diagnosis of asthma), and the hospital. All charges were adjusted to constant 2018 dollars using the Medical component of the Consumer Price Index [34,35].

Race or ethnicity: The HHIC race or ethnicity variable was based on the race or ethnicity categories that were consistently available across all Hawai‘i-based hospitals from January 2015 through December 2016. The HHIC performed regular quality assurance to assure racial or ethnic data were comparable across hospitals (e.g., clear data specifications, monthly reports to determine discrepancies, and regular meetings with HHIC provider stakeholders). The HHIC included only one primary race or ethnicity reported by each hospital, which was self-reported by parents or caregivers at the time of admission. Native Hawaiian, Japanese, Filipino, Chinese, other Pacific Islander, other Asian, and White populations accounted for more than 90% of the HHIC database. For this study, children from Native Hawaiian, other Pacific Islander, Japanese, Filipino, Chinese, White, and “other races” (combined category of American Indian, Black, Hispanic or Latino, other Asian, and other) populations were included. 

Covariates: Additional patient characteristics that may potentially confound the effects of PPPED visits were included. These were age, sex, comorbidity, insurance provider, location of residence, and location of hospital [14,23,28,36,37]. Comorbidities for asthma were based on the National Asthma Education Prevention Program (NAEPP), which included obesity, rhinitis or sinusitis, gastroesophageal reflux disease (GERD), allergic bronchopulmonary aspergillosis, obstructive sleep apnea, and stress or depression [22]. These were determined by ICD9/10 codes (see Appendix A). Sex was coded as male or female. Children were categorized into four-year age groups (e.g., 2–5, 6–9, 10–13, 14–17). Insurance providers were coded as public (Medicaid for disabled and low-income residents), private (including Hawai‘i Medical Service Association, Kaiser, other plans), Department of Defense (DoD), and other (uninsured or not otherwise specified). Locations of residence and hospital were categorized based on the state’s four designated counties (i.e., Hawai‘i, O‘ahu, Kaua‘i, and Maui).

### 2.4. Statistical Analysis

To describe the total cost burden by race or ethnicity, the total costs of all preventable ED visits for each race or ethnicity were summed. The average number of preventable ED visits by race or ethnicity was also calculated. 

The numbers of ED visits and costs of ED visits were compared by racial or ethnic group using the Kruskal–Wallis test due to the highly skewed distribution of cost data. Average costs across race or ethnicity adjusting for confounders were then predicted in a multivariable model using gamma regression with the log link function to avoid a retransformation bias and to correct for heteroscedastic errors [38]. Research has shown that gamma regression with log link is commonly used for cost data analyses because of its ability to address the potential for highly skewed distribution in cost data [39]. Predictors included in the model were race or ethnicity, age, sex, insurance provider, and location of residence. The cost ratios (CRs) and 95% confidence intervals (CIs) were calculated from the model and the goodness of fit was examined by using the residual deviance goodness of fit test [40].

All statistical analyses were performed using R version 3.4.1 from R Foundation for Statistical Computing in Vienna, Austria [41]. A two-tailed *p*-value of less than or equal to 0.05 was considered statistically significant. This study was determined to be exempt from human subjects review by the Institutional Review Board of the University of Hawai‘i at Mānoa.

## 3. Results

The total number of potentially preventable ED visits for pediatric asthma in Hawai‘i from 2015 to 2016 was 3230 visits by 2568 individuals, for a total cost of $1,912,454. Descriptive information about potentially preventable ED visits are in Table 2. The largest proportion of total potentially preventable ED visits came from Native Hawaiians (36.5%), followed by Filipino (19.4%), White (13.3%), other Pacific Islander (13.0%), other race (11.1%), Japanese (5.30%), and Chinese (1.5%).

Young children aged 2 to 5 years accounted for over 40% of PPPED visits, followed by children 6 to 9 years of age at 27%, children 10 to 13 years of age at 16%, and children 14 to 17 years of age at 17%. Males had higher percentages of PPPED visits (62.2%) than females. The majority of PPPED visits were paid by public sources (48.1%), with Medicaid/QUEST carrying the largest proportion. The average number of preventable ED visits per person was 1.26 (SD: 0.68).

Table 3 shows the descriptive data for PPPED visits per child by race or ethnicity. The average number of ED visits per child ranged from 1.19 to 1.29; however, the average number of ED visits per child did not vary significantly across racial or ethnic groups (*p* = 0.71). The average preventable ED visit costs varied significantly by race or ethnicity (*p* = 0.02). The groups with the higher median preventable ED visit costs were Japanese (median: $328; mean: $713), Chinese (median: $329; mean: $505), and Filipinos (median: $333; mean: $631). Other races (median: $298; mean: $674) and Whites (median: $298; mean: $495) had the lower median preventable ED visit costs. 

Table 4 shows the descriptive data for all PPPED visits by race or ethnicity. The groups with the highest median costs for all PPPED visits were Filipinos (median: $334; mean: $611) and Japanese (median: $332; mean: $687). Other races (median: $301; mean: $614) and Whites (median: $298; mean: $538) had lower median costs for all PPPED visits. There was also a wide range of PPPED visit costs seen within each racial or ethnic group. In most groups, the ranges varied from under $100 to $8000 for each ED visit. Visits from the “other race” group had the widest ED visit cost range, going from over $40 to over $10,000. Furthermore, the largest cumulative total cost burden over the study period was seen for Native Hawaiians at $572,985 and for Filipinos at $313,544. 

Table 5 shows the results of the multivariable model prediction costs, whereby the goodness of fit of the model indicates a proper fit (*p* = 0.89). No significant differences were seen for costs from preventable ED visits by race or ethnicity and sex. Age, insurance provider, and residency of the child were found to be significant predictors for costs from preventable ED visits. Children aged 10–13 years had a 22% higher (CR: 1.22; 95% CI: 1.02–1.45) average cost compared to children aged 2–5 years old. Children with DoD insurance had a 53% lower (CR: 0.47; 95% CI: 0.32–0.68) average cost compared to children with public insurance coverage. Additionally, children who resided on Hawai‘i island had a 42% lower (CR: 0.58; 95% CI: 0.49–0.69) cost compared to children who resided on O‘ahu; those who lived on Kaua‘i were 50% lower (CR: 0.50; 95% CI: 0.39–0.65), while those from Maui were 53% lower (CR: 0.47; 95% CI: 0.39–0.57). 

## 4. Discussion

This study compared the cost burden of potentially preventable ED visits for asthma-related services among Asian American, Pacific Islander, and White children using statewide data for Hawai‘i for the period 2015–2016. The largest cumulative and average costs were seen for Native Hawaiians, who had the largest proportion (36.5%) of all PPPED visits per child. Even though this finding could be attributed to Native Hawaiians being the largest racial or ethnic population in Hawai‘i, this was not seen for the other major racial or ethnic groups. For example, children of White descent are the second largest racial or ethnic group in Hawai‘i, and yet only accounted for 13.3% of all PPPED visits [2]. Furthermore, there were no differences in PPPED visits for children with at least one visit by race or ethnicity and sex. While we did not find racial or ethnic disparities in the cost and average number of PPPED visits, disparities in pediatric asthma prevalence are still of importance.

Our costs were only hospital costs, which did not account for indirect costs, including lost productivity among parents or caregivers, lost wages, school absenteeism, and premature death [42]. As a higher percentage of PPPED visits were Native Hawaiian, these ED visits may have a larger impact on children missing school and other childhood activities, as well as parents or caregivers missing work and reduced job productivity. This disparity highlights a significant and multifactorial burden for the Native Hawaiian community, who already experience social and health disparities. Native Hawaiians not only have the lowest levels of educational attainment, lowest household mean incomes, highest rates of poverty, and highest rates of environmental tobacco exposure, but Native Hawaiian children also have poorer health status compared to other racial or ethnic groups in Hawai‘i [4,43]. In order to reduce the economic burden on an already socially vulnerable population, resources should be allocated to community health partners that serve Native Hawaiian children by providing trustworthy, culturally grounded, and accessible asthma care. Research has shown culturally tailored programs may be a more appropriate approach for Native Hawaiians to improve and maintain health outcomes [44].

Asthma management and lowering the cost burden of PPPED visits are important policy issues, not only to address the economic burden on families, but also insurance payer spending. With the economic downturn of the COVID-19 pandemic, Medicaid is now covering a larger proportion of children across the US. Prior to the pandemic, Medicaid covered one third (33%) of children in Hawai‘i and was found to be the payer for a disproportionate amount of PPPED visits, at approximately 47% [45]. The multivariable analyses also showed the costs for public insurance payers were significantly higher than for private payers after adjusting for other factors. Given these findings, Medicaid should have a particular interest in developing initiatives that target pediatric asthma in Hawai‘i. 

Study findings showed that costs from PPPED visits were higher among children aged 10–13 years after adjusting for other factors. This could be attributed to the behavioral changes during adolescence, which can have a negative impact on a child’s asthma because of the decrease in adherence to treatment, with medical care becoming inconsistent [46]. Furthermore, this transition period is when a child’s cognitive abilities allow for more abstract thinking; therefore, asthma programs should take advantage of this critical transition period when adolescents have the cognitive capacity to assume responsibility and appropriately manage their asthma [10].

The findings also showed that PPPED visits were significantly lower among children from neighboring islands compared to those who reside on O‘ahu; however, there is a geographical maldistribution and lack of accessibility to ED facilities in rural areas and neighboring islands [36]. Families are forced to drive longer distances outside of their community to access the ED; therefore, the lower PPPED costs may be from families not seeking health care services because of the lack of access to ED facilities. 

The ineffectiveness of current health care practices to curb asthma-related outcomes among children highlights the need for asthma education programs. According to the NAEPP, effective asthma management includes educating children and families about the condition [22]. Asthma education provides more than basic information by combining knowledge that improves self-management skills and creates behavior change [22,47]. The education programs utilize various instructors, including nurses, clinicians, other health professionals, and community health workers [47,48]. Furthermore, asthma education programs can be conducted face-to-face or by telemedicine across a variety of settings, including hospitals, outpatient clinics, schools, and homes [15,22,48,49,50,51].

Many innovative programs have been implemented that specifically reduce asthma exacerbation events and the costs associated with acute care services (many of which are preventable) [23]. For example, in 1999, the Centers of Disease Control and Prevention (CDC) created the National Asthma Control Program, which funds health departments in 24 states and Puerto Rico to improve asthma management, treatment, and control in the US [52]. Hawai‘i is unfortunately not one of the states funded by the CDC, and there are no programs currently in place that target the high costs from preventable ED visits for those who are most impacted [53]; therefore, this study can help justify efforts to develop appropriate evidence-based asthma education programs for children from Hawai‘i in general, but specifically for children from Native Hawaiian populations who are between the ages of 10 and 13 and have Medicaid coverage. Even though the occurrence rates of asthma comorbidities were low and not significantly related to PPPED visits, asthma education programs should still address these medical conditions. The low prevalence of these comorbidities may be related to underdiagnosis or geographical location, but these comorbidities are nonetheless conditions (e.g., obesity, stress or depression) that impact the management of childhood asthma [54].

This study has strengths and limitations. One strength is that the HHIC data represent a census of all ED visits for Hawai‘i and not a sample, which allows for more reliable analyses with small numbers. Another strength includes the study sample size, with adequate numbers of understudied Asian American and Pacific Islander population groups across multiple EDs and payers; however, the study has limitations. First, the study sample started with children who had at least one PPPED asthma visit; therefore, identifying asthma disparities in PPPED visits among all children with asthma in Hawai‘i could not be estimated. Second, the cost estimates are based on ED facility charges. Even though these charges were adjusted using CCRs, this may not truly represent actual ED costs. Third, although there was an adequate number of individuals for each racial or ethnic group, they were relatively small and could have resulted in the insignificant findings by race or ethnicity found in the multivariable model. Fourth, there were also challenges with obtaining large enough sample sizes for preventable hospitalizations from each of the racial or ethnic groups to be able to produce statistically reliable analyses. Additionally, the HHIC data do not include data on physician office visits or other outpatient services that contribute to the overall direct cost burden from pediatric asthma. Fifth, other demographic factors, including educational attainment and household income of parents or caregivers, as well as pharmaceutical data, were not included in the HHIC database; therefore, these covariates could not be adjusted, which would have resulted in more reliable findings. Lastly, these cost estimates do not account for the indirect costs (e.g., loss of productivity from school absences and missed workdays by parents or caregivers) that also play a role in the financial burden associated with pediatric asthma.

Future research should include more detailed information from hospitalizations and outpatient data. These findings will provide a better understanding of the direct cost burden, highlight any racial or ethnic disparities, and focus efforts on policy changes for asthma management programs. Future research should also consider the indirect costs that also impact families and children with asthma, as these costs are expected to be larger. Children with asthma experience high rates of school absenteeism, which adversely impacts their academic success, while their parents or caregivers are forced to miss work or other activities to care for them, potentially impacting household income and their ability to financially afford asthma treatment [4,55]. Additionally, further research is needed to determine strategies that reduce risk factors for asthma among Native Hawaiian families, which include inadequate housing and stress, environmental tobacco exposure, lower educational attainment, and household income.

## 5. Conclusions

Acute care services, particularly ED visits for pediatric asthma, can be prevented. This study identified a large cost burden for potentially preventable ED visits in Native Hawaiian children; however, the costs per ED visit did not differ significantly by race or ethnicity. The economic burden from these preventable ED visits significantly impacted adolescent children and those with Medicaid. Further research is needed to better understand the overall direct and indirect cost burdens among children from the major racial or ethnic groups in Hawai‘i. Asthma programs that reduce potentially preventable ED visits could improve health inequities, as well as offset costs by government payers, hospitals, and families who have children with asthma.

## Figures and Tables

**Table 1:** Principal A\asthma ICD-9 and ICD-10 codes.

ICD-9 Codes	ICD-10 Codes
49300	J4521
49301	J4552
49302	J4522
49310	J45901
49311	J4531
49312	J45902
49320	J4532
49321	J45990
49322	J4541
49381	J45991
49382	J4542
49390	J45998
49391	J4551
49392	

**Table 2:** Descriptive information for potentially preventable ED visits for pediatric asthma from Hawai‘i Health Information Corporation Data, 2015–2016, using the first visit only (N = 2568).

	Total ED Visits n (%)
Race or ethnicity	
Chinese	39 (1.5%)
Filipino	497 (19.4%)
Native Hawaiian	936 (36.5%)
Japanese	136 (5.3%)
Other Pacific Islander	334 (13.0%)
Other Race	284 (11.1%)
White	342 (13.3%)
Age Group	
2–5	1036 (40.3%)
6–9	701 (27.3%)
10–13	403 (15.7%)
14–17	428 (16.7%)
Sex	
Female	970 (37.8%)
Male	1598 (62.2%)
Payer	
Public (Medicaid)	1234 (48.1%)
Private (HMSA, Kaiser, Private Other)	1101 (42.9%)
Department of Defense	73 (2.8%)
Other	160 (6.2%)
County (residence)	
Hawai‘i	455 (17.7%)
Kaua‘i	159 (6.2%)
Maui	310 (12.1%)
O‘ahu	1644 (64.0%)
County (hospital)	
Hawai‘i	459 (17.9%)
Kaua‘i	160 (6.2%)
Maui	309 (12.0%)
O‘ahu	1640 (63.9%)
Comorbidities	
Obesity	35 (1.4%)
Rhinitis/Sinusitis	17 (0.7%)
GERD	14 (0.6%)
Obstructive Sleep Apnea	3 (0.1%)
Stress/Depression	11 (0.4%)
	**Mean (** **±** **SD)**
Average # ED visits per person	1.26 (± 0.68)

Note. HMSA = Hawai‘i Medical Service Association; GERD = gastroesophageal reflux disease.

**Table 3:** Descriptive data for potentially preventable ED visits by individuals with asthma by race or ethnicity in Hawai‘i from Hawai‘i Health Information Corporation Data, 2015–2016 ^a,b^.

	Chinese	Filipino	Native Hawaiian	Japanese	Other Pacific Islander	Other Race	White
Total # of individuals	39	497	936	136	334	284	342
Average # of ED visits per child	1.28	1.20	1.29	1.19	1.25	1.29	1.27
Median Costs	$329	$333	$301	$328	$323	$298	$298
Mean (SD) Costs	$505 (±$801)	$631 (±$1118)	$612 (±$1103)	$713 (±$1340)	$600 (±$1153)	$674 (±$1356)	$495 (±$873)

^a^ Average costs (medians and means) by individuals (using data from first visit). ^b^ Costs are in constant 2018 dollars.

**Table 4:** Descriptive data for all potentially preventable ED Visits for Asthma by Race or ethnicity in Hawai‘i from Hawai‘i Health Information Corporation Data, 2015–2016 ^a^.

	Chinese	Filipino	Native Hawaiian	Japanese	Other Pacific Islander	Other Race	White
Total # of ED visits	50	598	1204	162	418	365	433
Median Costs	$315	$334	$306	$332	$326	$301	$298
Mean (SD) Costs	$476 (±$712)	$611 (±$1075)	$589 (±$1047)	$687 (±$1285)	$588 (±$1120)	$614 (±$1233)	$538 (±$969)
Cost Range	$110, $5196	$59, $8650	$47, $8429	$91, $7991	$28, $7287	$40, $10312	$47, $8265
Total Cost	$19699	$313544	$572985	$97031	$200449	$191556	$169401

Note: ^a^ Costs are in constant 2018 dollars.

**Table 5:** Multivariable generalized linear model with gamma distribution, log link function, and robust standard error estimation ^a^.

	2018 Inflation Adjusted Cost		
Predictors	Estimates (Cost Ratio)	95% CI	*p*
(Intercept)	691.29	555.53–860.21	<0.001
Race (ref: White)			
Chinese	0.89	0.53–1.48	0.65
Filipino	1.11	0.89–1.37	0.35
Native Hawaiian	1.17	0.97–1.43	0.11
Japanese	1.18	0.86–1.61	0.30
Other Pacific Islander	1.01	0.79–1.27	0.96
Other Race	1.19	0.93–1.52	0.16
Age (ref: 2–5 years old)			
6–9	1.07	0.92–1.24	0.39
10–13	1.22	1.02–1.45	0.03
14–17	1.18	0.99–1.40	0.07
Male (ref: Female)	0.90	0.79–1.02	0.09
Payer (ref: Public)			
Private	0.91	0.80–1.03	0.13
Department of Defense	0.47	0.32–0.68	<0.001
Other	0.92	0.71–1.18	0.51
Residency (ref: O‘ahu)			
Hawai‘i	0.58	0.49–0.69	<0.001
Kaua‘i	0.50	0.39–0.65	<0.001
Maui	0.47	0.39–0.57	<0.001

^a^ Data used from first visit only.

## Data Availability

Not applicable.

## Appendix A

**Comorbidity**	**ICD-9**	**ICD-10**
Obesity	2780 (except for 27803) 6491 79391 V853 V854 V8554	E6601 E6609 E661 E668 E669 O99210 O99211 O99212 O99213 O99214 O99215 R939 Z6830 Z6831 Z6832 Z6833 Z6834 Z6835 Z6836 Z6837 Z6838 Z6839 Z6841 Z6842 Z6843 Z6844 Z6845 Z6854
Rhinitis/Sinusitis	4778 4779	J302 J309
GERD	53081 53011	K21
Allergic bronchopulmonary aspergillosis	5186	B4481
Obstructive sleep apnea	32723	G4733
Stress/Depression	296 (except for 29680) 308 309	F43 F32 F33 F34
